# The calcium channel blocker Cav2.1 combined with amyloid beta monoclonal antibodies may reduce the occurrence of ARIA‐E

**DOI:** 10.1002/alz70859_096419

**Published:** 2025-12-25

**Authors:** Dinghao An, Xiaotong Li, Yun Xu

**Affiliations:** ^1^ Peking Union Medical College, Beijing, Beijing China; ^2^ Capital Medical University School of Basic Medical Sciences, Beijing, Beijing China; ^3^ Nanjing Drum Tower Hospital Clinical College of Nanjing Medical University, Nanjing, Jiangsu China

## Abstract

**Background:**

Amyloid‐targeting monoclonal antibodies were approved by FDA in the treatment of Alzheimer’s disease, some patients have experienced adverse reactions related to amyloid‐associated imaging abnormalities, including both cerebral edema‐type(ARIA‐E) and cerebral hemorrhage‐type(ARIA‐H), which significantly impact the overall prognosis of patients.

**Method:**

We proposed a hypothesis based on existing evidence from evidence‐based medicine and then evaluated it. We have preliminarily validated the therapeutic potential of the combination of Calcium channel blockers Cav2.1 and amyloid beta monoclonal antibodies in Alzheimer's disease on a point‐by‐point basis. In the future, we will conduct cell and animal experiments to validate its efficacy and safety. We will also investigate the mechanism of the combined use of the two drugs in improving cognitive function and reducing the occurrence of ARIA‐E.

**Result:**

Calcium channel blockers Cav2.1, such as Nimodipine, Nicardipine, dilate cerebral blood vessels, reduce both intracranial and extracranial pressure, and effectively penetrate and protect the blood‐brain barrier. These effects will maintain cerebral circulation and minimize neuronal damage. Calcium channel blockers Cav2.1, can promote vasodilation, allowing sufficient space in the vascular microenvironment for amyloid‐beta clearance. This allows for the rapid removal of released endogenous inflammatory factors, preventing their accumulation at specific sites on the blood vessel walls, which could otherwise lead to vessel wall obstruction. Additionally, by preventing the blockage of the brain's glymphatic system by amyloid fragments, it facilitates the removal of inflammatory factors and reactive oxygen species. Thereby, it could reduce mitochondrial dysfunction, endoplasmic reticulum stress, and rescue apoptotic or necrotic neurons.

**Conclusion:**

The use of calcium channel blockers Cav2.1, in combination with monoclonal antibodies may provide a new intervention strategy to reduce the occurrence and alleviate the symptoms of ARIA‐E.

